# Staged Repair of Concomitant Aortic Regurgitation and Descending Thoracic Aortic Aneurysm

**DOI:** 10.1055/s-0039-1679871

**Published:** 2019-03-08

**Authors:** Aamna Malik, Omar Nawaytou, Abdul Nasir, Deborah Harrington, Mark Field, Aung Oo, Manoj Kuduvali

**Affiliations:** 1 Medical School, University of Liverpool, Liverpool, United Kingdom; 2 Department of Cardiac Surgery, Liverpool Heart and Chest Hospital, Liverpool, United Kingdom

**Keywords:** aneurysm, thoracic aorta, left heart bypass, aortic valve insufficiency, airway obstruction, aortic valve replacement

## Abstract

Descending thoracic aortic (DTA) aneurysms causing left main bronchus compression can be surgically repaired under left heart bypass (LHB). Safe LHB requires a competent aortic valve. Some patients present with concomitant DTA aneurysms and severe aortic regurgitation (AR), precluding LHB as an adjunct for aortic surgery. The authors present such a case and outline the management. AR can safely be addressed first in an immediate staged surgical approach, providing adequate left ventricular function.

## Introduction

Compression of the left main bronchus is an uncommon complication of large aneurysms of the descending thoracic aorta (DTA). It requires urgent intervention and carries a risk of perioperative mortality. When concomitant aortic valvular pathology exists, urgent repair of both lesions presents a surgical challenge. Both the staging and timing of operation are crucial to achieving a successful outcome. We present a case of surgical aortic valve pathology coexistent with a DTA aneurysm complicated by left main bronchus compression. This report outlines a safe and effective method of managing the simultaneous conditions.

## Case Presentation

**Fig. 1:**
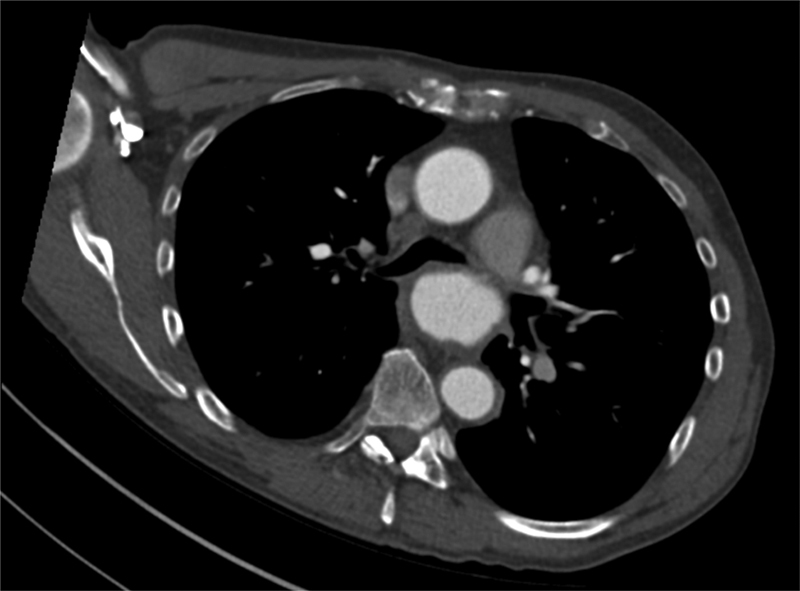
Transverse section of a contrast-enhanced computed tomography scan demonstrating compression of the left main bronchus measuring 2.4 mm at its narrowest.

**Fig. 2:**
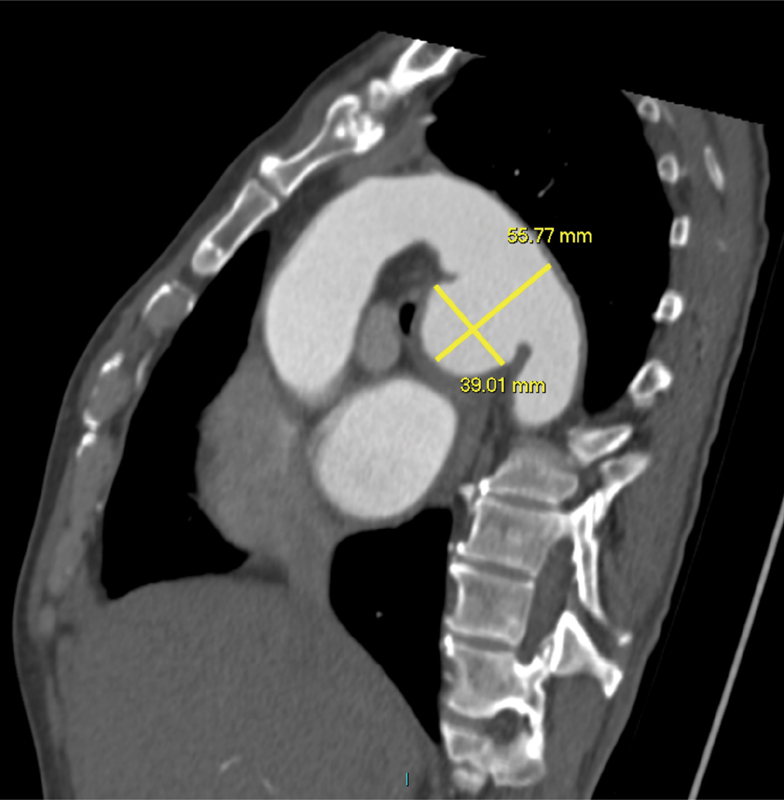
Sagittal section of a contrast-enhanced computed tomography depicting an anterior saccular aneurysm of the descending thoracic aorta with a maximum caliber of 56 mm.

A 52-year-old man presented to the general practitioner with 6-month history of atypical chest pain associated with dyspnea, hoarseness, and weight loss. On examination, the patient had stridor and was immediately referred to the acute medical unit. Computed tomography (CT) of his thorax showed a 6.5- × 4.5-cm DTA aneurysm effacing the proximal left main bronchus ( Fig. 1 ). A CT aortogram confirmed an anterior saccular aneurysm of the DTA with a maximum caliber of 5.6 cm, along with left main bronchus compression ( Fig. 2 ). There was no evidence of dissection or contrast leak. The patient was referred to our unit for urgent surgery.

On admission, a preoperative transthoracic echocardiogram revealed severe aortic regurgitation with tricuspid leaflets alongside moderate left ventricular (LV) dysfunction with an ejection fraction (EF) of 40%. His coronary angiogram was satisfactory with no flow limiting lesions; however, it demonstrated a right coronary artery (RCA) dominant system and a very short left main stem (LMS). Pulmonary function tests confirmed an obstructive defect (forced expiratory volume in 1 second [FEV _1_ ] 47% predicted, forced vital capacity [FVC] 66% predicted, FEV1/FVC 75%). His past medical history was significant for previously undiagnosed hypertension, empiric inhalers for symptomatic relief of dyspnea which were ineffective, and recent smoking cessation after a 15 pack year history.

The patient was taken to theater the day following admission for a planned mechanical aortic valve replacement (AVR) through a superior hemisternotomy followed by DTA repair through a left thoracotomy. Prior to sternotomy and heparinization for cardiopulmonary bypass, a spinal drain was inserted to reduce the risk of paraplegia during the anticipated second-stage DTA surgery. The patient was systemically cooled to 34°C and antegrade cold (4°C) blood cardioplegia was administered every 20 minutes. Cardioplegia was initially administered into the aortic root. However, severe AV regurgitation rendered this method inefficient; hence, it was later selectively administered into the left coronary artery and RCA ostia. Notably, the short LMS may have resulted in differential delivery down either the left anterior descending or left circumflex arteries. Owing to minimal access, it was not possible to administer retrograde cardioplegia.

Intraoperative findings suggested vasculitic changes including a blood-stained pericardial effusion with vascular adhesions and a thickened edematous ascending aortic wall. Intraoperative transesophageal echocardiogram showed global LV dysfunction with an EF of 30%.

The patient initially failed to come off bypass, so a second run of bypass was required to rest the myocardium and allow longer reperfusion as well as optimization of inotropic drugs and insertion of a pulmonary artery catheter. He was eventually weaned off bypass on moderately high inotropic and vasoconstrictor support (cardiopulmonary bypass time 145 minutes, aortic cross clamp time 108 minutes). Prolonged cross-clamp time was a result of limited access to the AV due to a small incision and a thickened aortic wall. The decision was made to delay his DTA repair until his LV function returned to his preoperative state and he was therefore transferred to the postoperative critical care unit.

On day 2 following AVR, a transesophageal echocardiogram showed recovery of LV function and the patient was taken back to theater for his second operation. Access was gained through a left fifth intercostal space thoracotomy, and he was put on left heart bypass (LHB) with one-lung ventilation to maintain distal aortic perfusion. The aneurysm was replaced with a rifampicin-impregnated 22-mm Dacron tube graft leaving the native aneurysm tissue adherent to the left main bronchus as a buttress. Motor evoked potentials were maintained throughout.

The remainder of his postoperative course was uneventful, and the patient was discharged home on postoperative day 7. At 6 weeks follow-up, he remains well.

## Discussion

Open repair is the procedure of choice for DTA aneurysms in young patients. This is best achieved using LHB to avoid pulmonary complications of full cardiopulmonary bypass, especially in the setting of left main bronchus compression and lung atelectasis. The complexity of this case arises from the fact that the patient had both left main bronchus compression necessitating urgent intervention on the DTA alongside concomitant severe aortic valvular regurgitation and LV dysfunction precluding LHB. Management options included a single-stage approach through a left hemi-clam shell incision, an immediate staged approach, a delayed staged approach, or a hybrid procedure.

A hemi-clam shell incision provides excellent exposure of the DTA and aortic valve, permitting a single-stage operation for concomitant DTA and cardiac lesions. 1 However, it is a major incision associated with prolonged and debilitating recovery. A single-stage repair requires full cardiopulmonary bypass and carries a significant risk of respiratory complications with prolonged mechanical ventilation and tracheostomy. In the setting of an atelectatic, potentially infected lung, LHB is more desirable in view of superior respiratory outcomes.

Although a hybrid approach with thoracic endovascular aortic repair may reduce surgical morbidity, it risks complete bronchial obstruction by increasing the pressure within the aneurysmal sac; prevention of this would require a left main bronchus stent that can be further complicated by the formation of an aortobronchial fistula. 2 Furthermore, surgery is preferable in young patients due to better long-term outcomes.

A staged operation is preferable to a single-stage clamshell incision due to lower risk of complications. A delayed staged approach is associated with high cumulative mortality, alongside risk of death from pulmonary complications in the interval between. In light of the clinical urgency of the situation, this patient was consented for an immediate staged repair through a superior hemisternotomy and lateral thoracotomy.

DTA repair is done under LHB to minimize the risk of spinal cord ischemia by maintaining distal aortic perfusion to the intercostal arteries. However, it requires adequate LV function alongside a competent aortic valve. Therefore, the AVR had to be done prior to the DTA repair. Aortic cross-clamping against a pumping LV in LHB results in sudden-onset hypertension proximally and afterload mismatch; in this case, it would have caused deterioration of the already impaired LV. 3

The patient had a significant reduction in LV function intraoperatively following AVR; thus, the decision to proceed immediately with the DTA operation was deemed inappropriate. Prolonging the operation, anesthesia, and cross-clamp time to complete the DTA repair would have significantly increased the risks of intraoperative morbidity and mortality. 4

This case demonstrates that open surgery can be safely performed as a delayed staged procedure with a short interval for concurrent aortic valve and DTA pathology. In a noncompetent aortic valve, AVR must be done prior to aneurysm repair of the thoracic aorta. Based on this case, implications may also be made for immediate staged repair providing the LV function is adequate.
